# Dysbiotic drift and biopsychosocial medicine: how the microbiome links personal, public and planetary health

**DOI:** 10.1186/s13030-018-0126-z

**Published:** 2018-05-03

**Authors:** Susan L. Prescott, Ganesa Wegienka, Alan C. Logan, David L. Katz

**Affiliations:** 1 School of Medicine, University of Western Australia, Princess Margaret Hospital, PO Box D184, Perth, WA 6001 Australia; 2 Department of Public Health Sciences, Henry Ford Hospital, Detroit, MI 48202 USA; 3 in-VIVO, Research Group of the Worldwide Universities Network (WUN), 6010 Park Ave, Suite #4081, West New York, NJ 07093 USA; 4 Prevention Research Center, Yale University, Griffin Hospital, 130 Division St, Derby, CT 06418 USA

**Keywords:** Microbiome, Health disparities, Ecology, Non-communicable diseases, Dysbiosis, Natural environments

## Abstract

The emerging concept of planetary health emphasizes that the health of human civilization is intricately connected to the health of natural systems within the Earth’s biosphere; here, we focus on the rapidly progressing microbiome science - the microbiota-mental health research in particular - as a way to illustrate the pathways by which exposure to biodiversity supports health. Microbiome science is illuminating the ways in which stress, socioeconomic disadvantage and social polices interact with lifestyle and behaviour to influence the micro and macro-level biodiversity that otherwise mediates health. Although the unfolding microbiome and mental health research is dominated by optimism in biomedical solutions (e.g. probiotics, prebiotics), we focus on the upstream psychosocial and ecological factors implicated in dysbiosis; we connect grand scale biodiversity in the external environment with differences in human-associated microbiota, and, by extension, differences in immune function and mental outlook. We argue that the success of planetary health as a new concept will be strengthened by a more sophisticated understanding of the ways in which individuals develop emotional connections to nature (nature relatedness) and the social policies and practices which facilitate or inhibit the pro-environmental values that otherwise support personal, public and planetary health.

## Background

“*Even with all our medical technologies, we cannot have well humans on a sick planet. Planetary health is essential for the well being of every living creature. Future healthcare professionals must envisage their role within this larger context, or their efforts will fail in their basic objective. Although until recently healthcare providers could ignore this larger context, such neglect can no longer be accepted*” [1].

Thomas Berry, 1992.

The term planetary health, popularized in the 1980s–1990s, underscores that human health cannot be uncoupled from the health of natural systems within the Earth’s biosphere. In their 1991 textbook on biopsychosocial medicine, psychologists Judith Green and Robert Shellenberger underscored that “*planetary health is not separate from our own*” [2]. More recently, the Lancet Commission on Planetary Health published its keystone report [3]; they concluded that political, economic and social systems – the policies and practices which define modernity - intersect with all life on planet Earth. Specifically, planetary health was formally defined in this context as “*the health of human civilization and the state of the natural systems on which it depends*”, and one of the primary goals of the planetary health concept is to find ‘*solutions to health risks posed by our poor stewardship of our planet*’ [3]. The Report acknowledged that the path to planetary health must run through a greater understanding of human behaviour in the context of social, psychological and biological influences.

The biopsychosocial paradigm is concerned with the simultaneous attendance to biological, psychological, and social dimensions of illness [4]. As such, biopsychosocial medicine is intimately connected to the ecological theatre in which individuals accumulate their life experiences while interacting with other ‘actors’ - fellow humans and other forms of life on Earth [5]. Global biodiversity - that is, the variety of species, their genetic contribution, and the ecosystems they form - is essential for the promotion of human health and well-being, including mental health [6, 7]. However, the clinical relevancy of this reality often escapes discourse in the context of biopsychosocial medicine. So, too, researchers in the new realm of planetary health may overlook the importance of the biopsychosocial paradigm, privileging technology and biomedicine in its discourse (as evidenced by the absence of the terms ‘psychosocial’ or ‘biopsychosocial’ in the 56-page Lancet Commission on Planetary Health Report).

While not as readily visible as other species in the natural environment, such as the Giant Panda or Giant Sequoia, microorganisms are the unseen form of life in the ecological theatre that can underscore the importance of planetary health for human health. Microorganisms may be best suited to illuminate both the ways in which exposure to biodiversity supports health, and the ways in which stress, positive and negative emotions and social polices interact with lifestyle and behaviour to influence the biodiversity that mediates health. While biological diversity is a long recognised feature of healthy environments, a wealth of new data now reveal how microbial ecosystems sit at the foundations of the many, diverse natural systems which sustain human health [8].

Here in our Commentary, we focus on the emerging microbiome science which serves to underscore the importance of accumulated experiences within the total lived environment, and how these experiences push upon biological systems; microbiome science conveniently unifies each portion of the biological, psychological and social equation which is so critical to personal, public and planetary health. We underscore at the outset that despite the remarkable advances in the science of the micobiome, the body of work remains largely in its infancy. At this stage, scientists have yet to discover an ‘ideal’ microbiome (although strides are being made in identifying gut microbial signatures which separate health and disease [9]), and indeed much of the research is in the realm of correlation, not causation [10]. However, there is enough microbiome research in place to allow this ‘unseen’ form of nature - potentially influenced by psycho-social conditions, societal policies and practices - to help erase the lines between biopsychosocial medicine and planetary health.

**Fig. 1:**
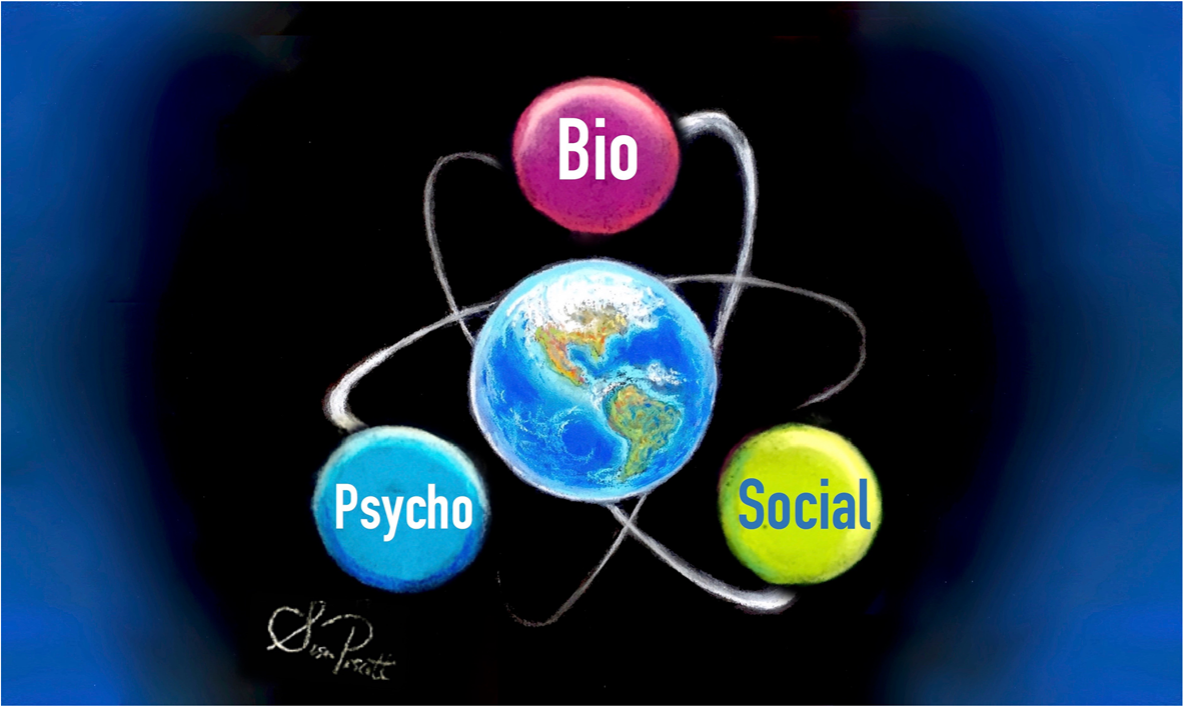
Human Health as Dependent Upon Planetary Health, BioPsychoSocial Inputs as Determinants of Planetary Health

Specifically, we will argue that microbiome science is illustrating that biopsychosocial medicine and the emerging planetary health paradigm are essentially one-in-the-same; discussions of one necessitates discussions of the other because the ‘ecosystems’ of political and social systems shape the ecological theatre (including the unseen microbes within urban or natural environments) which shapes us (Fig. 1). In order to support that argument, we first explore some of the history and context of microbiome science, and then turn toward the ways in which psychosocial factors can influence the human microbiome in the modern environment. These include stress, dietary patterns, contact with natural environments and overall lifestyle. Finally, we discuss pathways to unify discussions of biopsychosocial medicine and the emerging paradigm of planetary health.

## Microbiome - history and context

In 1988, scientists working in the field of plant ecology defined the microbiome “*as a characteristic microbial community occupying a reasonably well defined habitat which has distinct physio-chemical properties. The term thus not only refers to the microorganisms involved but also encompasses their theatre of activity*” [11]. Now, three decades later, the microbiome has become a core focus of virtually every branch of science and medicine; emerging research is shifting the historical vantage, a place from which microbes were viewed largely, if not exclusively, as a pathogenic threat. Despite positive effects on morbidity and mortality, it has now become clear that successes in the reduction of infectious diseases via non-specific antibiotics has, at least in part, involved collateral damage to beneficial microbes. Further, widespread antimicrobial use in animal rearing practices and unnecessary prescribing in clinical settings may have untold consequences to the ecosystems large and small [8, 12].

In many ways, this unseen microbial world – some 1 trillion species in the biosphere [13] and up to 100,000 microbes on a single grain of sand [14] – is now illuminating distinct and considerable bridges of connectivity between all life forms on Earth. For example, disturbances in microbial ecology are revealed to underpin shifts in soil, plant, animal and human health with modern industrialisation and agricultural practices, including the widespread use of antibiotics, pesticides, and other chemicals [8, 12, 15]. At the same time, research suggests that the application of beneficial microbes can improve soil health, promote the growth of nutrient-dense plants [16], detoxify environmental pollutants [17] and even buffer against colony collapse disorder in bees by protecting against the immunotoxicity of pesticides [18].

Disturbances to the complex commensal microbial communities - that is, loss of beneficial microorganisms, and/or the expansion of potentially harmful microbes, and/or the loss of overall microbial diversity - is termed dysbiosis [19]. In the context of planetary health, it is interesting to note that the Greek etymological origin of dysbiosis translates as ‘difficult living’ or ‘life in distress’; given the contemporary pressures of climate change, environmental degradation, grotesque health inequalities, non-communicable disease (NCDs) epidemics, biodiversity losses, and rapid urbanization, we have argued that the original meaning of dysbiosis is apropos [20]. As we will describe in more detail below, dysbiotic drift is a term used to describe the ways in which the westernized environment pushes microbial dysbiosis (and ‘life in distress’) in non-random ways; that is, it pushes more forcefully on a gradient slanted toward socioeconomic disadvantage [21].

In the context of biopsychosocial medicine, the microbiome is upending how we view the human ‘self’; from the biological perspective, it is no longer tenable to view ourselves as functionally separate from the organisms that live on and within us [22]. As we accumulate our life experiences - growing, learning, working, playing and loving - our *Homo sapien* protein-coding genes are massively outnumbered by the microbial genes we carry [23]. Importantly, the microbiome contributes functional genes which influence many aspects of human physiology, including those of the nervous system. Thus, examining the biological ‘self’ means looking through the lens of the holobiont; this term refers to the multicellular eukaryote and the inseparable colonies of persistent symbionts which together form a critically important unit of anatomy, physiology, immunology, growth, and evolution [24]. Together, the human host and its microbiome (microbiota and their collective genomes) are an ecological community existing in an ecological theatre.

## Microbiome, form and function

Throughout healthy natural systems, microbiota contribute to ‘ecosystem services’; they play a role in events ranging from cloud formation [25] to the protection of plants in the presence of insect predators [26]. For humans, the microbiome is essential in the maintenance of barriers to the external environment (e.g. cutaneous structures and intestinal mucosa), normal ‘training’ of the developing immune system, protection against pathogens, and the metabolism of xenobiotics. In addition, the microbiome sits at the interface of nutrition and metabolism; functions include nutrient extraction, production of vitamins, transformation of dietary phytochemicals, lipid metabolism, provision of short chain fatty acid (and a host of other potentially bioactive metabolites) [5, 27, 28].

Increasingly, metagenomic and other comparative human studies reveal reduced biodiversity and compositional alterations of the gut and skin microbiota are associated with various inflammatory NCDs, including asthma, allergic and inflammatory bowel diseases, type 1 and type 2 diabetes, obesity, depression and other many other NCDs [29]. Added to this, there is strong evidence that a significant component of the risk of many of these NCDs is programmed in early life – even those that do not manifest for decades [30], forming the basis for the developmental origins of health and disease (DOHaD) hypothesis [31]. As with all bodily systems, the immature infant microbiota is more vulnerable to environmental pressures, paradoxically during a period when per capita antibiotic usage is most intensive [12]. Countless experimental animal models provide confirmatory data and elucidate mechanistic pathways, including how early life NCD risk factors (stress, nutrition, antibiotics, toxins and environmental biodiversity) can mediate their effects through the developing microbiome [32]. This suggests that, as with infectious disease, the prevention and treatment of NCD may also depend on optimising microbial ecology, albeit in a different way.

This also draws obvious focus to the immune system at the core of all interactions between the external environmental and the internal body systems [32]. The immune system is critically dependent on microbial exposure for its initial maturation and ongoing function, and dysbiotic changes in both gut [20] and skin microbiota [33] yield immune dysregulation and an abnormal propensity for inflammation [20]. Indeed, aberrant inflammation is the common pathogenic link between altered gut microbiota and the diverse array of diseases contributing to the NCD burden, including mental ill-health. This underscores the central role of the immune system mediating the multisystem consequences of dysbiosis as well as strategies that might be employed to overcome it.

The threat of pathogenic microorganisms, of course, still remains; parasites, soil-transmitted helminths such as hookworms, large roundworm, and whipworm, still represent a critically important causes of chronic morbidity in the context of planetary health [34]. On the other hand, the pathogenicity of these organisms appears to be determined, at least in part by gut bacteria [35], and the declining exposure to helminthic parasites and harmless environmental saprophytes in westernized nations may compromise immunoregulation. This ‘deficiency’ of exposure (relative to our ancestral past) has been linked to increased risk of various non-communicable diseases [36]. In other words, our challenge is to find the ‘sweet spot’ of controlled exposures so that we derive benefit from a robust ‘endobiome’ (that is, the microbial world inside us, and inside the planetary biosphere), while avoiding adverse exposures. That invites a return to ‘nature,’ but a modified nature that does not threaten us routinely with malaria and harmful parasites [37].

## Microbiome, the brain and stress

The first clue that microbes have an important role in brain physiology dates back to a landmark study in 1986; here, researchers showed differences in brain histamine levels between conventional and germ-free animals [38]. Researchers also showed that miniscule amounts of orally administered *Campylobacter jejuni* activated the visceral sensory nuclei in the brainstem and promoted anxious behavior in animals [39]; the mechanism of this gut microbe-induced brain activity was possible through direct gut-to-brain communication via the vagus nerve [40]. In addition, other groups were demonstrating that various forms of stress - heat, cold, acoustic, crowding, physical exhaustion, restraint, food deprivation, maternal separation - could disrupt the normal gastrointestinal microbiota in animals; this led to early suggestions that the clinical administration of beneficial microbes might have positive effects in physical and cognitive fatigue and improve depressive symptoms [41, 42].

The wellspring of contemporary gut-brain-microbiota interest is often traced to the landmark (2004) research of Nobuyuki Sudo and colleagues; this team found that brain derived neurotrophic factor (BDNF) gene expression was lower in the hippocampus and the cortex of germ-free animals compared with conventionally-raised specific pathogen-free animals. Given the role of BDNF in nerve plasticity, it was a clear indication that commensal microbes could influencing brain structure and function. Moreover, Sudo’s team also demonstrated enhanced hypothalamic-pituitary-adrenal axis activity among germ-free animals following acute stress, thus adding even more strength to the idea that microbiota are involved in programming the stress response [43]. Separate research groups have also used germ-free and specific pathogen-free animal models to confirm that early-life microbial colonization initiates signaling mechanisms that impact the neuronal circuits involved in motor control and anxiety behavior [44].

The pathways by which the microbiome might influence cognition and mental health are not completely understood, and the reader is referred to elegant papers which review possible mechanisms in detail [45, 46]. Briefly, the microbiota–brain–gut axis is thought to communicate via neural routes (the vagus nerve in particular, which carries microbially-mediated information to the brain [47]), humoral signaling molecules (e.g. cytokines), neuropeptides and hormonal messengers. Gut microbes influence the integrity of the intestinal barrier, which, if compromised, can initiate a cascade of low-grade inflammation and metabolic dysregulation [48]. In addition, the gut microbiome is central to the realm of nutritional psychiatry; gut microbes act upon dietary components ranging from amino acids (e.g. tryptophan the serotonin precursor) to polyphenols (producing bioactive metabolites) which are directly and indirectly capable of influencing mood [49, 50].

Volumes of animal studies have since accumulated on the effects of stress as a disturbing factor to the microbiome; even short-term social stress has been shown to disturb the mammalian microbiome [51, 52]. These animal studies are supported by human research which has linked perceptions of psychological stress, depressive symptoms and anxiety with microbial dysbiosis [53–56]. In addition, patients with major depressive disorder and other mental disorders have been noted to have intestinal microbiome profiles which differ from healthy controls [57–62]. Moreover, early life antibiotic exposures [63] and repeated antibiotic use in adolescents and adults [64] have been linked to subsequent depression. Even aspects of personality such as conscientiousness have been linked to microbiome signatures in cross-sectional research [65].

Although the links between human stress and alterations to the microbiome are correlational, and the functional meaning (if any) of these microbiome differences in human depression/anxiety is an area of strong scientific interest [66], there is experimental evidence which argues for at least some degree of causality. For example, there is evidence from non-human animals providing a strong argument that once dysbiosis is in place, the altered microbiome compounds depression, anxiety and/or cognitive dysfunction. For example, when researchers transplant faecal material from human donors with depression or anxiety into healthy recipient rodents, these animals display behaviours indicative of depression and anxiety. These behavioural changes did not occur when animals were the recipients of faecal material from healthy human donors [67–69].

The mechanisms by which stress can cause dysbiosis are not completely understood; the presence of stress hormones can directly influence the growth of select microorganisms and indirectly influence microbial adhesion to mucosal surfaces [70]. Stress can promote the production of inflammatory signaling chemicals which subsequently influence dysbiosis, and it can change gastrointestinal motility, gastric secretions, and other aspects of gastrointestinal physiology [71, 72]. Studies in rodents and humans shows that stress impacts dietary choices; often this translates into the consumption of highly palatable, energy-dense, nutrient-poor, additive-rich foods [73–76]. In turn, these are the dietary choices - low in fiber, phytochemicals and essential fatty acids - which are implicated in gut microbiome dysbiosis [77, 78].

## Biomedical solutions

Unsurprisingly, the emerging gut microbiome-brain research has resulted in much enthusiasm for biomedical therapeutics which aim to manipulate the microbiome for mental and neurological benefit [79, 80]; these include the administration of probiotics (living microbes that when administered in adequate amounts, confer benefit to the host [81]), prebiotics (a substrate that is selectively utilized by host microorganisms with a resultant health benefit [82]) or encephalo-biotics (non-viable microbes, microbial parts and/or other agents that influence the microbiome with resultant benefits in cognition, mental well-being or brain health [31]). Certainly, there is encouraging research from preliminary human intervention studies indicating that live [83–85] and even heat-inactivated microbes [86–89] can positively influence mood, stress, anxiety and sleep. There is also evidence from human studies indicating that fermented food consumption (rich in fatty acids and probiotics) is associated with lower anxiety/depressive symptoms [90, 91], improved immune function under mental stress [92, 93], and changes in brain activity suggestive of potential value in reducing reactivity to stressful stimuli [94].

The reader is referred to expert reviews specifically focused on the therapeutic possibilities of biological, microbe-based interventions on human mental wellbeing; undoubtedly there is much promise here. However, in the discourse concerning the microbiome and mental health, the social context is often ignored. Some scholars have questioned the extent to which the biomedical-dominated microbiome narrative masks the persistent, underlining psychosocial and ecological drivers of distress, mental disorders and precariousness which contribute to dysbiosis in both its broad meaning, and its microbial definition. In the race toward microbiome-targeting biomedicines, it is necessary to underscore that he holobiont does not exist in a vacuum; the saliency of the ecological theatre in the mental health-microbiome discourse is made clear by examining the environmental factors that have been linked to microbial dysbiosis (in human and/or animal studies) in westernized nations and how these overlap with socioeconomic disadvantage (the populations with highest risk of distress, depression and mental disorder).

## Social context, disadvantage and Dysbiosis

Undoubtedly, there are many biopsychosocial factors which can influence health and longevity, and there is much to be learned from nations with notable longevity such as Japan and Sweden (as well as from affluent nations such as the United States that do not enjoy high-ranking longevity) [95, 96]. However, within westernized-industrialized nations, socioeconomic disadvantage is well known to increase the risk of mortality. Disadvantage may ‘get under the skin’ and manifest, biologically, as allostatic load (the cumulative ‘wear and tear’ of responses to stress) [97, 98]. We argue that disadvantage in socioeconomic position also ‘gets into the gut’.

Since an individual’s gut microbiome is largely a product of environmental exposures rather than genetic inheritance, the psychosocial aspects of the microbiome discourse are critically important [99]. As mentioned above, the most obvious provocateur of microbial dysbiosis is the insidious western dietary pattern with its ultra-processed foods, refined fats and excess sugar [100]. The absence of colorful fruits and vegetables and whole grains compounds dysbiosis by its deficiency in specific nutrient and phytochemical intake (e.g. polyphenols in whole plant foods, magnesium in green leafy vegetables and natural nitrate found in vegetables) [101–103]. In addition, the westernized diet contains food additives – sodium, emulsifiers, artificial sweeteners, phthalates, pesticide residues – that have each been linked to dysbiosis in humans or animal models [104–110]. Further, the ways in which the modern westernized foods are prepared – using high-heat in the absence of water (e.g. roasting, baking, frying) is increasing the presence of advanced glycation end-products (AGEs) which have been tied to inflammation, oxidative stress and microbial dysbiosis [111, 112].

The dominant presence of highly-processed foods displaces fresh fruits and vegetables as well as traditional fermented foods, and in doing so limits the intake of beneficial microbes that would otherwise be carried with the latter two groups of foods [113, 114]. The quest to feed the most people for the lowest cost has reduced starvation; however, the continued focus on food production as an industry rather than as a public health intervention now pushes nutritional content lower. Perhaps not surprisingly, child obesity and ultra-processed food consumption have simultaneously increased [115, 116].

However, there are other lifestyle factors implicated in microbial dysbiosis. Excess alcohol consumption, sedentary behavior, improper sleep or disturbances in circadian rhythms, and tobacco exposure have been linked to dysbiosis [117–120]. In addition, other exposures – including those at the neighborhood level - are also implicated; environmental pollutants including airborne particulate matter, lead, mercury, polycyclic aromatic hydrocarbons and phthalates have been linked to intestinal dysbiosis [110, 121, 122]. Also included on the list of pathways to dysbiosis, perhaps the most obvious, is antibiotic and antimicrobial exposure [12]. It is important to underscore that these are almost exclusively single-exposure studies; however, the emerging exposome science suggests that there will be a synergistic and/or cumulative dysbiotic response to these collective exposures over time [32, 123, 124]. The cumulative exposures which may erode diversity of the human microbiome in westernized nations - and its link to a higher burden of NCDs in SES disadvantaged individuals and communities - has been described as the ‘dysbiotic drift’ hypothesis [21].

The dysbiotic drift theory pulls the lens back from single exposures; when examining the risk factors for dysbiosis it becomes evident that these are the very same factors related to the total lived experience in socioeconomic disadvantage. Psychological stress, westernized diet (with its missing nutrients and added chemicals, AGEs) consumption, circadian disruptions, excess alcohol and tobacco use, phthalate and environmental chemical exposure, and higher antibiotic prescriptions are not randomly distributed. These factors of the exposome press upon the microbiome along SES lines [21, 31]. Indeed, mucosal biopsy samples (one of the more accurate ways to sample the intestinal microbiome) demonstrate reduced diversity of the intestinal microbiome among residents of disadvantaged neighbourhoods in North America [125].

In sum, a mix of pre-clinical and human studies demonstrate that the very same lifestyle factors that are connected to NCDs are interconnected with disturbances to the microbiome. While the composition of the ‘ideal microbiome’ is yet unidentified, and likely to remain so in the near-term, there is enough research to suggest that once in place, dysbiosis may amplify the risk of NCDs. Again, the impact of dysbiotic forces are no doubt greater in early life before a mature microbiome has been established. The early-life application of microbial biomedicine such as probiotics may hold the greatest promise as an intervention for subsequent, later-life mental health and healthy cognitive function [126]; however, the dominance of biomedical solutions, and marketing of those remedies, can obscure upstream ‘causes of the causes’ and ignore opportunity for prevention of dysbiosis.

Thus, biomedicine is attempting to undo dysbiosis (life in distress) by manufacturing drug-oriented solutions for the problem of microbial dysbiosis; at the same time, the problem of both dysbiosis (life in distress) and microbial dysbiosis is being manufactured, at least in part, by a system in which the multinational marketing of dysbiotic products is left unchecked. Available evidence shows that marketing effectively influences childhood dietary choices [127]. It also shows that those dietary choices are interwoven with multiple lifestyle factors - screen time, sleep, stress, and environmental availability of food choices. As stated succinctly in *The Lancet* (2013), profits in the ultra-processed food, alcohol and tobacco industries encourage pandemics of NCDs [128]. Experts in biopsychosocial medicine must assess to what extent dysbiosis in the modern environment is sustained by the marketing of unhealthy choices and an overall lifestyle which is at odds with the prevention of NCDs [129–135] and to what extent do social policies maintain or mitigate the maintenance of dysbiosis, especially along socioeconomic lines.

## Planetary context

Returning to the concept of planetary health - the interconnectivity of the health of human civilization and that of natural systems - the microbiome science revolution is at once an important area for research and clinical consideration, and a metaphor for a broken system. Microbiome science underscores the decades-old message of biopsychosocial medicine - that the patient in the waiting room, whether in attendance for a wellness, preventative, diagnostic or treatment visit - is a manifestation of the total environment in which they live, and have lived [32].

Based on research involving the dwindling populations of those living in relative isolation from westernization and urbanization, our hunter-gatherer ancestors - as well as those living an early subsistence lifestyle - likely lived with a far more diverse microbiome. With a good degree of consistency, international studies involving hunter-gatherers and/or communities that remain relatively isolated from westernization have shown higher levels of gut and skin microbial diversity [136–138]. Without the advantages of antimicrobials and vaccines, these groups are less well protected against pathogens and early-life threats. However, it is now possible to theorize that microbial diversity mediated by lifestyle plays a role in later life-course NCD resilience among such groups [139]. Experimental studies suggest that losses in microbial diversity associated with westernized lifestyle might be a product of diminished intake of certain dietary constituents, most notably fibre and phytochemicals [140, 141].

Notwithstanding the possibility of uniquely tailored dietary plans for specific conditions, the preponderance of evidence indicates that dietary patterns which favour human health are those which support gut microbial diversity (or at least, specific shifts in bacterial dominance linked to health) [142]; in turn, diets rich in healthy plant foods and limited in animal products are also those which appear to lessen the burden of greenhouse gas emissions, environmental degradation and other threats to planetary health [143–147]. For example, estimates through to 2050 suggest that global expansion of western-style dietary patterns rich in animal products would lead to massive increases (80%) in greenhouse gas emissions and require up to 740 million hectares of additional cropland (compared with a healthy diet modelled between the Mediterranean, pescetarian and vegetarian diets) [147].

In the bonds between biopsychosocial medicine and planetary health, we further underscore that the microbiome isn’t exclusively a gut and diet story. Emerging research shows that exposure to microbes associated with outdoor natural environments may also have health-promoting properties, particularly in early life in association with training the immune system. No longer is the intact dermis considered a fortress wall impenetrable to microbes; experimental evidence suggests that skin microbes may have systemic immune activity which opens the door to links between microbes in the total lived environment and many aspects of health [33].

If cutaneous microbes have a systemic influence, it magnifies the importance of the ways in which urbanization and socioeconomic position links to the skin microbiome and changing microbiomes within homes and residential areas [148, 149]. Residential proximity to trees and other aspects of natural environments has been linked to mental health [150]. There are some hints that this may be, at least partly, mediated by immune-microbiota interactions via by contact with microbes found in natural environments [16, 151–155].

Understanding the ways in which experience with nature, especially early in life, influence subsequent attitudes toward nature (which, in turn may determine certain pro-social and pro-environmental behaviors) is a critical research objective in the realm of planetary health. The extent to which an individual values the natural world in their daily life is measureable. For example, validated scales of nature relatedness (also nature connectivity, nature connectedness) collate individual awareness of, and fascination with, the natural world; nature relatedness and the related scales assess the degree to which an individual has an interest in making contact with nature. Of importance to biopsychosocial medicine and planetary health, nature relatedness has been linked with psychological wellbeing, empathy, pro-environmental attitudes and humanitarianism (and negatively with materialism) [156–159].

The sum of existing research indicates that life course experience with (and early perceptions of) nature can shape nature relatedness and pro-environmental attitudes/behaviors; since previous research published in *BioPsychoSocial Medicine* [160] and elsewhere [161] indicates that westernization and urbanization is coincident with increasing psycho-emotional disconnect from nature, researchers are actively exploring which types of environments (and activities therein) can elevate nature relatedness and shape environmental attitudes that either promote or detract from personal, public and planetary health [162–167]. For example, emerging research shows that children who frequently experience nature are likely to develop greater emotional affinity to and support for protecting biodiversity [168]. Moreover, understanding specific types of human-nature relationships may help predict pro-environmental behaviors [169].

Once again, this emerging research necessitates discourse concerning social and ecological considerations; if green space and contact with natural environments is a health asset, do all citizens have equitable access to nature and opportunity to develop a psychological connection to the Earth’s natural systems? The available research suggests that not only does socioeconomic disadvantage push dybiosis from one direction in the form of stress, ultra-processed foods, pollution etc., it also does so by absentia; disadvantage is often associated with less green space access and opportunity to make contact with nature. Moreover, lack of a living wage and the need to work additional hours (or even two jobs) with minimum compensation does little to open up time for recreation in natural environments [31]. Time for outdoor recreation may be an asset associated with affluence [170].

Thus, pairing limited time with limited financial resources may reduce an individual’s ability to choose foods and living environments that are likely to increase personal microbial diversity. When individuals are faced with providing nourishment for themselves and their dependents, while under financial duress, there is only an illusion of food choices as these individuals are often limited to selecting from a wide array of inexpensive, highly palatable, ultra-processed, high refined-fat and low-fibre foods that promote dysbiotic drift. Further, changing diet and activity patterns at individual and family levels requires time, energy and patience – something in short supply in societies most heavily affected by shrinking microbial diversity. These are complex discussions which include socio-political-economic ideologies and the systems that maintain uneven power dynamics in the context of biopsychosocial medicine [171].

These interconnected issues of high complexity need to be explored from a variety of avenues; new interventional birth cohorts, such as *Born in Bradford* [172], the *ORIGINS project* [173], the *ECHO* project [174], and a growing network of prevention intervention cohorts which are now examining the influence of many of these environmental domains on improving health outcomes within an ecological frame work: local community projects contributing to the planetary health narrative. The goal is to explore wider protective and buffering factors that enhance resilience and reduce allostatic load, such as building nature relatedness, interpersonal relationships, purpose, mindfulness and positive emotions and sleep. This will determine whether these ‘upstream’ approaches to wellness behaviour will have flow on effects to the ‘usual’ risk targets (such as poor nutrition, physical inactivity, stress and substance abuse) by influencing these core behaviours through better relationships with self, community and the environment. It is our hope that in addition to scientific pursuit, that community cohorts, especially those focused on young families, could be part of the solutions in *every* community - actually nourishing individuals and whole communities towards positive change. In essence, cohorts studies represent a pathway for global change to begin locally – the opportunity to engage as part of an interconnected grass-roots strategy to understand the complexities which can help promote global health.

## Conclusion

Personal health, and indeed that of human civilization at large, is coupled to the health of natural systems within the Earth’s biosphere. While biological diversity is a long recognised feature of healthy environments, a wealth of new data now reveal how microbial ecosystems sit at the foundations of the many, diverse natural systems which sustain human health [8]. Over the last century, human activity and modern lifestyle changes have had a major impact on ecosystems, large and small, with many of the adverse consequences mediated through disturbances of microbial ecosystems [8]. This includes effects on the human microbiome – now known to have a critical influence on most aspects of health and development, with epigenetic and even transgenerational effects [8]. These perspectives underscore the need for an integrated, ecological framework when considering the human health and environmental challenges of the twenty-first century. The emerging discipline of planetary health is a multi-sectoral effort which recognises the interconnectivity of personal and public health with thriving ecosystems, and the need for urgent systemic solutions to address the health of the natural environment and our relationship with it [3]. As such planetary health cannot be uncoupled from biopsychosocial medicine, and vice versa [5]. In this, it is crucial that resulting policies and societal decisions are made with a comprehensive understanding of the nexus between lifestyle, the microbiome and human health.

With advances in research, it is realistic to hope that the microbial contribution to healthy ecosystems, spanning macro to micro scales can be leveraged for personal, public and planetary health to provide solutions to some of our most pressing problems, including our ability to sustain the life that nourishes and sustains humanity. In the twentieth century, scientific medicine began its very successful campaign against infectious disease, reducing mortality and improving quality of life through innovations in antimicrobial development, vaccines and a variety of public health strategies, including anti-hunger programs. However, now the health pendulum has swung such that so-called NCDs are now the leading cause of mortality worldwide and disproportionally affect the most vulnerable [175], indicating that the herd now needs to be protected in other ways.

As we have outlined, planetary health *is* biopsychosocial medicine. The individual consuming critical public health information or sitting in the ‘waiting room’ is at once a product of the health of the planet, and a significant determinant of planetary health. We have used the emerging microbiome science - acknowledging its current limitations - to illustrate how an ‘unseen’ form of nature can illuminate the connections between biopsychosocial medicine and planetary health. While ‘manipulating the microbiome’ with advances in biomedicine may soon help to prevent and treat NCDs, there is also a need to tackle the causes of global dysbiosis. The etymological root of dysbiosis means ‘life in distress’, and a rebalancing of the scales of psychosocial - to match the privileged dominance of biomedicine - may be a early priority of the growing planetary health movement.

## Abbreviation

NCDs: non-communicable diseases
